# Development and Validation of a Machine Learning Prediction Model for Textbook Outcome in Liver Surgery: Results From a Multicenter, International Cohort: Erratum

**DOI:** 10.1097/AS9.0000000000000613

**Published:** 2025-11-03

**Authors:** Jane Wang, Amir Ashraf Ganjouei, Taizo Hibi, Nuria Lluis, Camilla Gomes, Fernanda Romero-Hernandez, Han Yin, Lucia Calthorpe, Yukiyasu Okamura, Yuta Abe, Shogo Tanaka, Minoru Tanabe, Zenichi Morise, Horacio Asbun, David Geller, Mohammed Abu Hilal, Mohamed Adam, Adnan Alseidi

In the article: *Development and Validation of a Machine Learning Prediction Model for Textbook Outcome in Liver Surgery: Results From a Multicenter, International Cohort* by Wang et al., which was published in *Annals of Surgery Open* on February 10, 2025, the following author name was spelled incorrectly:

Zeniche Morise, MD, PhD

The correct reference is:

Zenichi Morise, MD, PhD

The authors apologize for the oversight.
